# A case of complete response to radiotherapy combined with durvalumab and tremelimumab in a patient with unknown primary hepatocellular carcinoma arising in the lumbar spine

**DOI:** 10.1007/s12328-024-02044-4

**Published:** 2024-10-24

**Authors:** Aiko Tanaka, Tomokazu Kawaoka, Shinsuke Uchikawa, Hatsue Fujino, Atsushi Ono, Eisuke Murakami, Clair Nelson Hayes, Daiki Miki, Masataka Tsuge, Shiro Oka

**Affiliations:** 1 Department of Gastroenterology, Graduate School of Biomedical and Health Sciences, Hiroshima University, 1-2-3, Kasumi, Minami-ku, Hiroshima, 734-8551 Japan; 2 Liver Center, Hiroshima University Hospital, Hiroshima, Japan

**Keywords:** Hepatocellular carcinoma, Radiation therapy, Durvalumab, Tremelimumab

## Abstract

A 58-year-old man visited an orthopedic clinic complaining of pain in his right lower back and numbness in his lower limbs for one month. Imaging tests revealed a tumorous lesion from the left side of the second lumbar vertebra to the paraspinal muscles. CT-guided biopsy of the tumor was performed, and immunostaining results diagnosed hepatocellular carcinoma (HCC). Although the liver showed signs of chronic liver damage, no primary tumor was found within the liver or in other organs. Blood tests showed negative hepatitis virus markers for both HBV and HCV. The tumor markers AFP, AFP-L3, and DCP were high. Because he developed spinal cord compression syndrome due to a lumbar tumor, radiation therapy and denosumab administration were performed. Subsequently, systemic therapy with durvalumab plus tremelimumab was started. In the year following the start of treatment, the tumor has shrunk, and no new lesions have been observed. Tumor markers have also decreased. We have experienced a case of HCC in the lumbar spine without a primary tumor in the liver. This is a very rare case, and the combination therapy with durvalumab and tremelimumab resulted in a complete response, which we consider to be a valuable case.

## Introduction

HCC is the sixth most common cancer worldwide and the third deadliest [1], and patients with hepatocellular carcinoma are frequently treated. Hepatocellular carcinoma originates in the liver, but there are rare types of HCC that arise outside the liver. These may originate as ectopic hepatocellular carcinoma, hepatoid adenocarcinoma, or spontaneous necrosis of intrahepatic hepatocellular carcinoma. Treatment of hepatocellular carcinoma includes liver resection or liver transplantation, local therapy, chemoembolization, and systemic drug therapy with durvalumab and tremelimumab or atezolizumab and bevacizumab, with immune checkpoint inhibitors being the first-line systemic drug therapy. In recent years, it has become known that the combination of radiation therapy and immune checkpoint inhibition can enhance therapeutic efficacy [2]. In this report, we describe a case of a very rare HCC of unknown primary that was successfully treated with radiotherapy and an immune checkpoint inhibitor (ICI).

## Case report

**Fig. 1:**
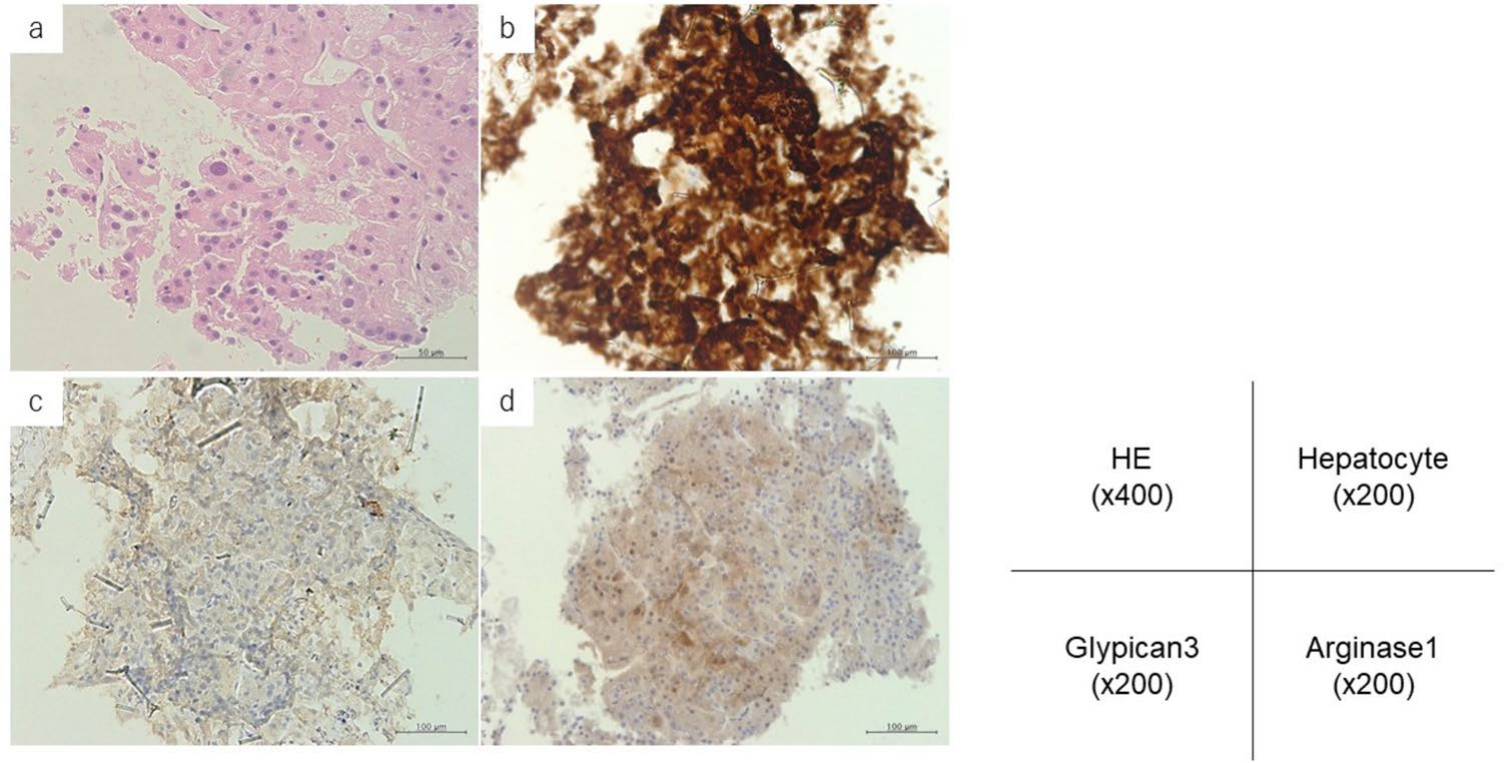
Histopathological features of the tumor located in the second lumbar vertebrae. **a** Hematoxylin–eosin staining (HE) (× 400). **b** Hepatocyte marker is positive in cytoplasm (× 200). **c** Glypican3 marker is negative in cytoplasm (× 200). **d** Arginase1 marker is positive in the cytoplasm (× 200)

The patient is a 58-year-old male on hemodialysis attending a local physician for type 2 diabetes mellitus and end-stage renal failure. In December 202X, he visited an orthopedic surgeon because he was aware of right lumbar pain and numbness in his lower limbs. Lumbar spine MRI showed a 4 cm neoplastic lesion from the left side of the second lumbar vertebral body to the paraspinal muscles. The patient was referred to our hospital in February 202X + 1 for close examination of lumbar spine tumor. The results of CT-guided aspiration cytology showed Class V, and immunostaining showed Hepatocyte-positive, Glypican-3-negative, Arginase1-positive, and S100-negative histology, leading to the diagnosis of HCC (Fig. 1).

Contrast-enhanced CT and MRI were performed to search for the primary tumor. The liver showed evidence of chronic liver damage, but no primary tumor was found in the liver or other organs, and no obvious primary tumor was noted on upper and lower gastrointestinal endoscopy.

PET-CT showed an accumulation of SUV max 8.8 only in the lumbar spine tumor area. Based on imaging and pathology results, he was diagnosed as cT0N0M1, cStage IVB, BCLC stage C HCC.

Blood tests at the initial visit showed T-Bil 0.8 mg/dL, AST 19U/L, ALT 21U/L, Alb 3.8 g/dL, PT activity 96%, and liver function was preserved. He was also HBsAg negative, HBsAb negative, HBcAb negative, HCVAb negative, anti-nuclear antibody negative, anti-mitochondrial antibody negative, and his background liver was NBNC. Tumor markers included elevated AFP 57.2 ng/mL, AFP-L3 11.5%, DCP 261mAU/mL (Table 1).

**Table 1:** Laboratory examinations

Complete blood cell count		
WBC	9410	/μL
Neutrophil	66.4	%
Lymphocytes	19	%
Monocytes	8.8	%
Eosinophil	5.3	%
Basophil	0.5	%
RBC	4.43 × 10^4^	/μL
Hb	12.5	g/dL
Ht	39.6	%
Plt	146 × 10^3^	/μL
Blood coagulation test		
PT activity	96	%
PT-INR	1.01	
Blood chemistry		
T-Bil	0.8	mg/dL
AST	19	IU/L
ALT	21	IU/L
LDH	190	IU/L
ALP	100	IU/L
γ-GTP	46	IU/L
Na	137	mEq/L
K	4.1	mEq/L
Cl	100	mEq/L
TP	7.4	g/dL
Alb	3.8	g/dL
BUN	31.6	mg/dL
Cr	10.2	mg/dL
CRP	0.07	mg/dL
NH3	29	μmol/L
HbA1c	6.2	%
Viral markers		
HBs antigen	(–)	
HBs antibody	(–)	
IgGHBc antibody	(–)	
HCV antibody	(–)	
Tumor markers		
AFP	57.2	ng/mL
AFP-L3	11.5	%
DCP	261	mAU/mL
CEA	4.3	ng/mL
CA19-9	60.6	U/mL
Immunology		
ANA	< 40	
AMA	< 1.5	

WBC: white blood cells, RBC: red blood cells, Hb: hemoglobin, Ht: hematocrit, Plt: platelet, T-Bil: total bilirubin, AST: aspartate aminotransferase, ALT: alanine aminotransferase, LDH: lactate dehydrogenase, ALP: alkaline phosphatase, γ-GTP: gamma-glutamyltransferase, TP: total protein, Alb: albumin, BUN: blood urea nitrogen, Cre: creatinine, CRP:C reactive protein, Na: sodium ion in blood, K:potassium ion in blood, Cl: chlorine ion in blood, BS: blood sugar, HbA1c: hemoglobin A1c, PT: prothrombin, HBs Ag: hepatitis B surface antigen, HBs Ab: hepatitis B surface antibody, HBc Ab: hepatitis B core antibody, HCV Ab: hepatitis C virus antibody, AFP: alpha-fetoprotein, DCP: des-γ-carboxy prothrombin, CEA: carcinoembryonic antigen, CA19-9:carbohydrate antigen 19–9, ANA: anti-nuclear antibody, AMA: anti-mitochondrial antibody

**Fig. 2:**
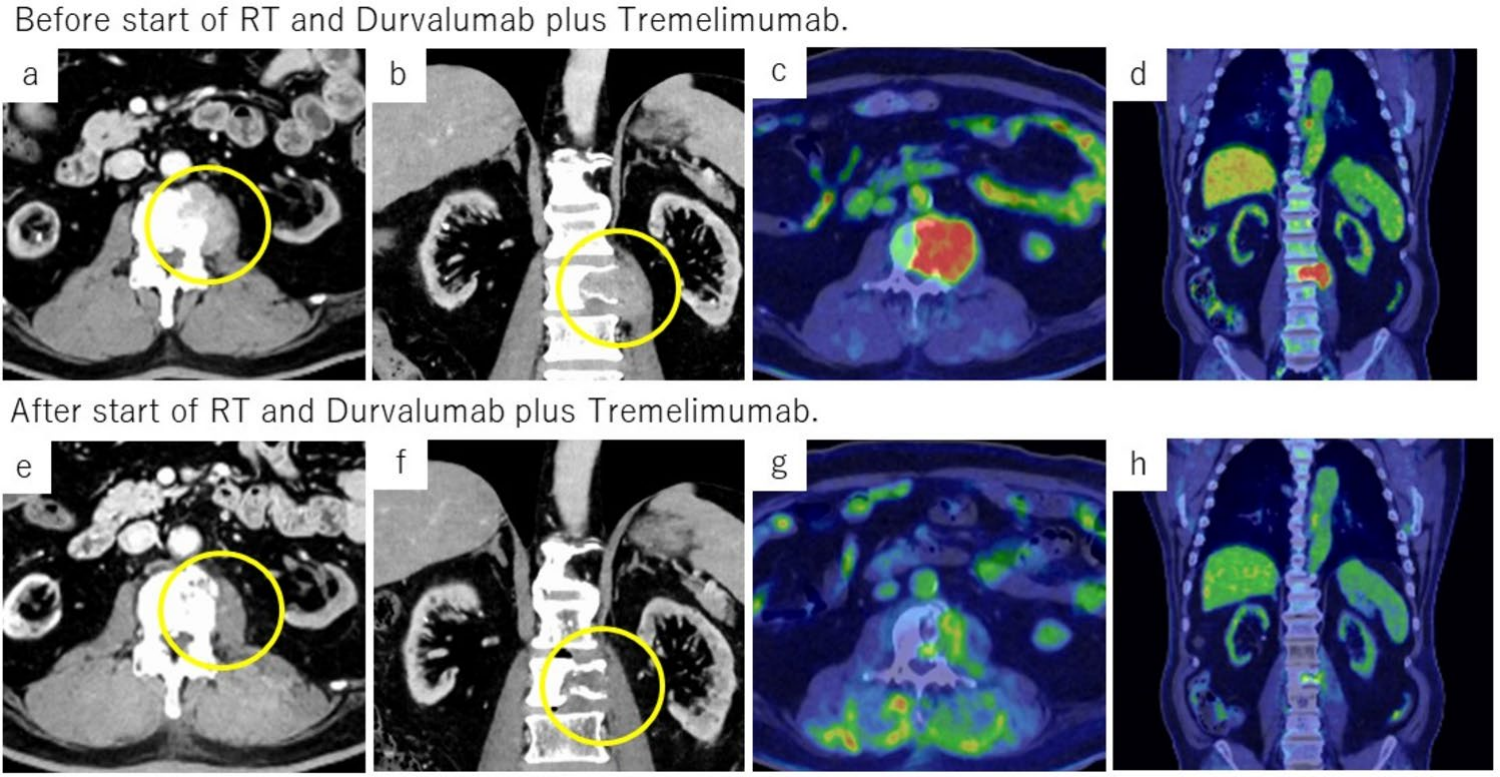
Contrast-enhanced computed tomography (CT) imaging test and Fluorine-18-fluorodeoxyglucose positron emission tomography (FGD-PET) imaging test results before and after treatment. **a**–**d** Before RT and durvalumab plus tremelimumab. **e**–**h** 9 months after RT and durvalumab plus tremelimumab, 8 courses of durvalumab

**Fig. 3:**
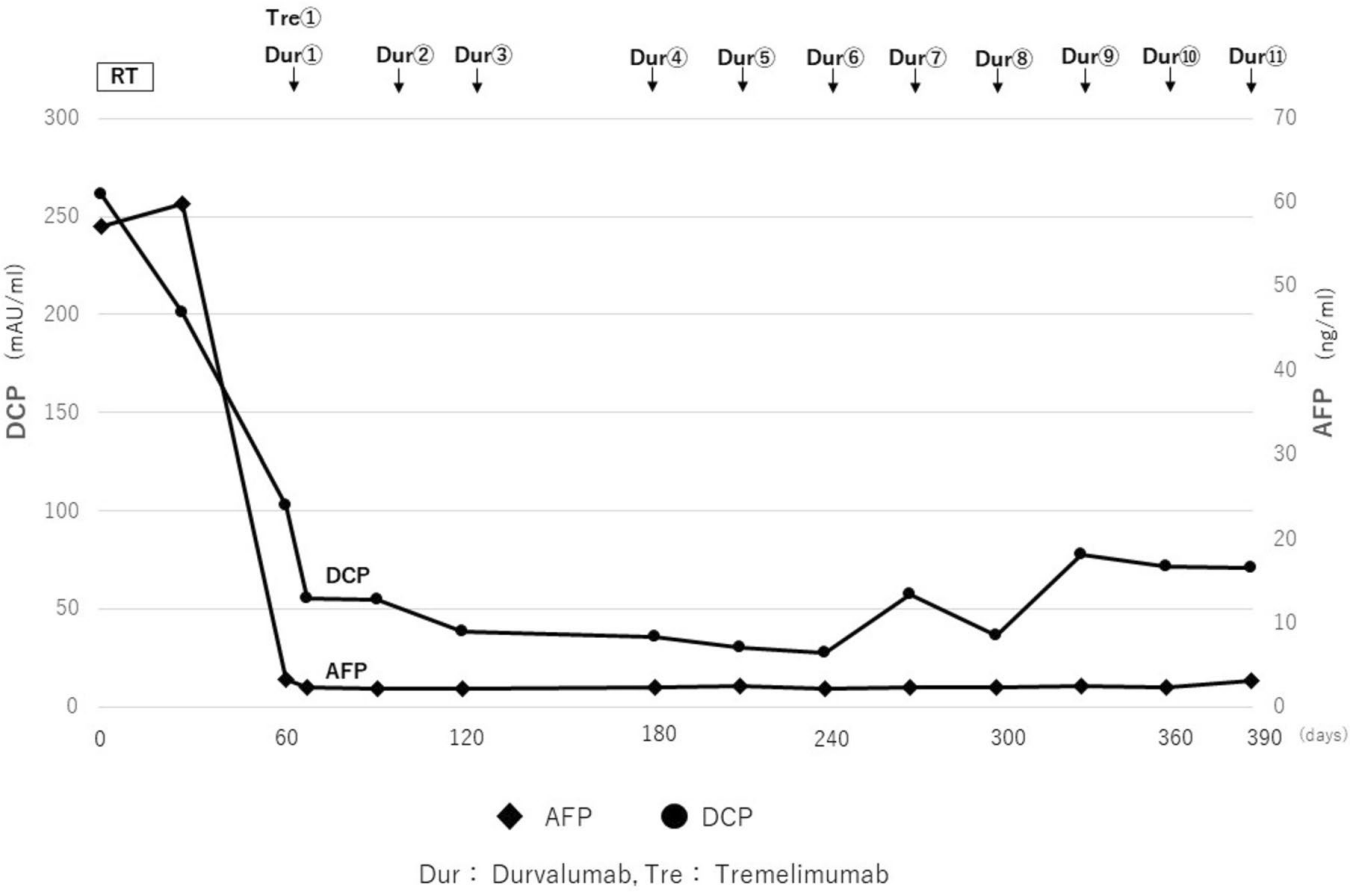
Clinical course and trend in tumor markers of the patient. After radiotherapy (39 Gy/13fr) and durvalumab plus tremelimumab administration were started, tumor markers (AFP and DCP) decreased and remained low for a long time

Two weeks after diagnosis, the patient had worsening numbness and pain in both lower extremities at the time of consultation. He was diagnosed as having spinal cord compression syndrome due to a lumbar tumor, and was urgently hospitalized and treated with 39 Gy/13 Fr of radiation and denosumab. After completion of radiotherapy, his symptoms improved, and his AFP, AFP-L3, and DCP levels were confirmed to have decreased (Fig. 2). On day 28 after completion of radiotherapy, a combination of durvalumab and tremelimumab was started as systemic drug therapy. As of April 202X + 2, a total of 11 courses have been administered since the start of treatment, but tumor markers have remained low, and imaging studies have not revealed any new lesions. Contrast CT and PET-CT showed that the lumbar spine tumor had shrunk, SUVmax was 3.3 (Fig. 3), the accumulation was reduced, and the mRECIST CR was maintained for a long period of time. During treatment, adverse events included hypertension, diarrhea, pruritus, and fatigue, all CTCAE (Common Terminology Criteria for Adverse Events) (version5.0) Grade 1, which improved only with symptomatic treatment.

## Discussion

Primary liver cancer is the sixth most frequent cancer in the world and the third most common cause of cancer-related death worldwide [1]. This is a very rare case of HCC arising in the bone without a primary tumor in the liver.

When HCC tissue is found only outside the liver, the differential includes ectopic hepatocellular carcinoma with a primary tumor, hepatoid adenocarcinoma of bone origin, and bone metastasis of HCC without a primary tumor.

Ectopic hepatocellular carcinoma is defined as HCC arising from the liver parenchyma located in extrahepatic organs or tissues [3], Some case reports of ectopic hepatocellular carcinoma have been reported in patients without liver diseases such as viral hepatitis or cirrhosis, and the mechanism of carcinogenesis is believed to be different from that of HCC arising in normal sites, which is caused by biliary obstruction or vascular insufficiency of ectopic hepatocytes [4]. The sites of ectopic HCC have been reported to include the gallbladder, pancreas, omentum, retroperitoneum, and adrenal glands, and are thought to be most common around the liver and gallbladder [5]. Although there have been no reports of ectopic hepatocellular carcinoma arising in bone, a case of hepatocellular carcinoma with a primary site in the thoracic spine was reported based on the finding that bone marrow-derived cells are the origin of hepatocellular carcinoma [6]. The possibility that this case is also an ectopic hepatocellular carcinoma with a lumbar spine as the primary site cannot be ruled out.

Hepatoid cell carcinoma is a tumor with features of hepatocellular carcinoma and adenocarcinoma arising in organs other than the liver [7]. It is known to occur in various organs, including the stomach, colon, lungs, pancreas, and uterus, but there are no reports of its occurrence in bones [8]. In this case, upper and lower gastrointestinal endoscopy revealed no neoplastic lesions in the gastrointestinal tract, and CT showed no neoplastic lesions other than in the lumbar spine. Pathologically, there were no findings suggestive of hepatoid adenocarcinoma, and the possibility of bone-derived hepatoid adenocarcinoma was considered unlikely.

Next, we will discuss the case of a bone lesion being a metastatic lesion of hepatocellular carcinoma. The most common sites of HCC hematogenous extrahepatic metastasis are lungs (55%), abdominal lymph nodes (41%), bones (28%), adrenal glands (11%), and peritoneum (11%) [9]. The most common site of bone metastasis of HCC is the spine, followed by the pelvis and ribs [10]. In general, extrahepatic metastases usually occur in patients with advanced HCC [11]. Case reports of hepatocellular carcinoma with bone metastases but no primary tumor in the liver at the same time are very rare. I found the 15 cases of HCC in bone without a primary lesion in the liver by searching for previous reports. [12–25] (Table 2).

**Table 2:** cases of extrahepatic HCC without a primary lesion in the liver identified by literature search and the our case

Case	Author	Year	Age/sex	Tumor lesion	AFP (ng/ml)	HBs Ag	HCV Ab	Diagnostics
1	Raoul [12]	1995	58/F	Skull	66,000	(–)	(–)	Operation
2	Horita [13]	1996	61/M	Sternum	4464	(–)	(+)	Operation
3	Iosca [14]	1998	71/M	Lt iliac bone	Normal	Untested	Untested	Operation
4	Asselh [15]	2001	66/M	Lt chest wall	Normal	(–)	(–)	Operation
5	Hofmann [16]	2003	61/M	Rt chest wall	Normal	(–)	(–)	Operation
6	Coban [17]	2004	71/M	Lt chest wall	60,000	(+)	(–)	Biopsy
7	Qureshi [18]	2005	50/M	Sternum	18,302	(–)	(–)	Biopsy
8	Hyun [19]	2006	51/M	Lt chest wall	308	(–)	(+)	Operation
9	Khalbuss [20]	2007	62/M	Lt chest wall	16,425	Untested	Untested	Aspiration
10	Takahama [21]	2010	63/M	Lt iliac bone	114.6	(–)	(–)	Biopsy
11	Jung [22]	2012	74/M	Rt iliac bone	Normal	(+)	(–)	Biopsy
12	Koh [23]	2014	46/M	Lt chest wall	292	(–)	(–)	Biopsy
13	Hwamg [24]	2015	61/M	Cervical spine	5013.1	(+)	(–)	Biopsy
14	Abbas [25]	2015	54/M	iliac bone	430	(–)	(+)	Biopsy
15	Shirota [6]	2022	82/M	Thoracic spine	Normal	(–)	(–)	Biopsy
16	Our case		58/M	Lumbar spine	57.2	(–)	(–)	Biopsy

The absence of a primary tumor in the liver may be due to spontaneous regression of the hepatocellular carcinoma or metastasis from an HCC too small to be detected by imaging studies.

Spontaneous regression of cancer is estimated to occur in 1 case in 60,000 to 100,000 [26], and spontaneous regression of HCC is reported to occur in 1.1% to 2.4% of cases. The main mechanisms of spontaneous regression of hepatocellular carcinoma are necrosis due to ischemia and impaired blood circulation and activation of tumor immunity. The former, necrosis due to ischemia and impaired blood circulation, is thought to be caused by secondary nutrient vessel occlusion and poor angiogenesis due to catheterization such as abdominal angiography, damage to the lining of nutrient arteries, circulatory disturbance due to vessel occlusion, massive bleeding, impaired blood flow due to shock, and rapid tumor growth [27, 28]. The latter activation of tumor immunity has been attributed to the infiltration of inflammatory cells into the tumor, capsule, and surrounding tissues and the associated anti-tumor effects of elevated cytokines such as TNF-α. Triggers that have been cited include external factors such as blood transfusion, trauma, surgery, biopsy, drug involvement, abrupt alcohol cessation, alcoholism, smoking cessation [29], etc.

It is also possible that bone metastasis may have developed from HCC of a size that was difficult to detect in imaging studies. In the present case, bone metastasis occurred at the stage of microscopic HCC, which could not be identified by imaging at the time of initial diagnosis, and intrahepatic lesions may have disappeared or spontaneous necrosis may have occurred due to systemic drug therapy.

Treatment options for HCC include hepatectomy or liver transplantation, local therapy, chemoembolization, and systemic pharmacotherapy, but no standard treatment has been established for extrahepatic HCC without a primary tumor. The patient was diagnosed with stage cT0N0M1, cStageIVB, BCLC stage C. According to Western and Japanese guidelines [30, 31], first-line treatment in this situation is systemic drug therapy. Drug therapy includes the use of molecularly targeted agents and immune checkpoint inhibitors alone or in combination. Currently, durvalumab and tremelimumab, atezolizumab and bevacizumab, lenvatinib, and sorafenib are commonly used as first-line therapies for metastatic HCC. The patient underwent radiotherapy (39 Gy/13fr) to palliate symptoms of spinal cord compression due to bone metastasis, followed by systemic drug therapy with durvalumab and tremelimumab according to the guidelines.

The patient has been able to maintain CR for a long period of time, approximately one year, which may have been due to the combination of radiotherapy and ICI. In recent years, it has become clear that irradiation of malignant tumors elicits a specific anti-tumor immune response that enhances therapeutic efficacy. Irradiation of malignant tumors releases an immunostimulatory protein (HMGB1), which activates dendritic cells. In addition, T cells are activated when dead cancer cells are recognized and presented as cancer antigens through phagocytosis by dendritic cells [32]. The PD-1 receptor is an immune checkpoint receptor expressed on the surface of activated T cells that induces peripheral immune tolerance by binding to PD-L1 [33, 34]. Radiation induces PD-L1 expression in cancer cells and immune tolerance. This indicates that irradiation activates anti-tumor immunity, while at the same time the tumor reinforces immune escape mechanisms [32]. Thus, the combination of radiotherapy and PD-1/PD-L1 inhibitors is expected to enhance the therapeutic effect. On the other hand, CTLA-4 is an immune mechanism regulatory molecule expressed on the surface of activated T cells and constitutes an inhibitory pathway called the immune checkpoint; blocking CTLA-4 has been reported to enhance antitumor immunity [33, 34]. Based on the above, we believe that the combination of radiotherapy and the immune checkpoint inhibitors durvalumab and tremelimumab has suppressed tumor growth and maintained CR over the long term.

## Conclusion

We have experienced a case of HCC in the lumbar spine without a primary tumor in the liver. This is a very rare case, and the combination of durvalumab and tremelimumab resulted in a complete response, making this a valuable case. In addition, the combination of radiotherapy and immune checkpoint inhibition may be useful in enhancing the therapeutic effect.

## Abbreviations

HCC: Hepatocellular carcinoma
AFP: α-Fetoprotein
DCP: *des*-γ-Carboxy prothrombin
HBV: Hepatitis B virus
HCV: Hepatitis C virus
NBNC: Non-hepatitis B virus, non-hepatitis C virus
ICI: Immune checkpoint inhibitor
CT: Computed tomography
MRI: Magnetic resonance imaging
PET-CT: Positron emission tomography–computed tomography
SUV: Standardized uptake value
BCLC: Barcelona Clinic Liver Cancer
RECIST: Response Evaluation Criteria in Solid Tumors
CR: Complete response
CTCAE: Common Terminology Criteria for Adverse Events
